# Neonatal seizures therapy: we are still looking for the efficacious drug

**DOI:** 10.1186/1824-7288-39-37

**Published:** 2013-06-05

**Authors:** Carlotta Spagnoli, Elena Pavlidis, Francesco Pisani

**Affiliations:** 1 Child Neuropsychiatric Unit, Neuroscience Department, University of Parma, Parma, Italy

**Keywords:** Neonatal seizures, Therapy, Antiepileptics, Anticonvulsants, Newborns

## Abstract

Therapeutic options currently available for neonatal seizures are still unsatisfactory both in terms of efficacy and of risk for long-term neurotoxicity, even if there is growing recognition of their potential to worsen neurodevelopmental outcome. A recent paper by Slaughter and colleagues entitled “Pharmacological treatment of neonatal seizures: a systematic review” has been published with the aim to provide a treatment algorithm, but, due to the relative paucity of clinical studies, it relies mainly on traditional antiepileptic drugs and does not distinguish between different neonatal populations, especially preterm and hypothermic neonates, who might require a dedicated approach in order to improve seizure control and reduce side effects.

## Background

Neonatal seizures therapy is still a controversial issue in neonatal neurology as drug options are limited and disappointing, whereas failure to control them with antiepileptic therapy has been associated with a worsening in subsequent outcome [1], urgently prompting the implementation of shared protocols.

The effects of neonatal seizures on the immature brain are difficult to distinguish from those of the brain lesions causing them, however the presence of prolonged seizures in the neonatal period represents an important risk factor for poor prognosis or developing neurological sequelae [1]. With an estimated prevalence of clinical seizures varying from 1/1000 to 3/1000 live births and even higher rates among preterm babies, depending on gestational age and birth weight, from 4.4/1000 live births between 1500 and 2500 g, 55-130/1000 live births <1500 g and up to 64/1000 live births in infants <1000 g birth weight [2], this is a relevant medical problem. Moreover, in spite of a reduced mortality (nowadays ranging from 7% to 16%) in term babies, preterm birth carries a high mortality risk (22-58%), and the overall risk of impairment following neonatal seizures is still as high as 30% [2].

Despite the high potential risk of adverse neurological outcome in newborns with prolonged seizures, there are currently no efficacious therapies.

### Main text

Slaughter et al. [3] have recently published an exhaustive systematic review of trials and observational peer-reviewed studies focusing on the pharmacological therapy of neonatal seizures, in which they offer a very comprehensive discussion on drug regimens, responsiveness, time needed to stop seizures, side effects, aims and duration of EEG monitoring.

In view of the available evidence a treatment algorithm is proposed, starting with IV phenobarbital as the first-line drug (possibly to be repeated once) followed by either IV levetiracetam, phenytoin/phosphenytoin or lidocaine as second-line and, in case of refractoriness, a trial of pyridoxine followed by IV midazolam. Last options considered include pentobarbital coma or lidocaine (unless previous administration of phenytoin/phosphenytoin).

## Discussion

### Traditional antiepileptic drugs

Thus, very little has changed and first-line and in part second-line drugs have remained the same for decades, in spite of the generally unsatisfactory effectiveness of traditional antiepileptic drugs, the little evidence to support the use of any of them over the other [4] and the increasing recognition of their potential neurotoxicity, linked to enhanced neuronal apoptosis [5,6]. Phenobarbital is still considered as the first-line approach due to longer clinical experience and the inclusion in randomized controlled trials on neonatal seizures therapy [7]. The only partial efficacy of conventional antiepileptic drugs might at least in part derive from the existence of an unbalance between excitatory and inhibitory pathways due to age-specific ion-channels expression and function, as suggested by animal models [8], although still not yet demonstrated in the human newborn, making immature brain unique and efficacy of drugs adopted from adult brain physiology potentially questionable.

### New perspectives

Unfortunately, these notions on pathophysiological peculiarities of developing brain have not yet been translated into a change in clinical practice due to the lack of robust evidence in human newborns. Theoretically bumetanide use seems the most promising strategy, due to its mechanism of action being specifically directed against NKCC1, which is developmentally expressed and responsible of GABA-mediated excitation in the newborn animal brain [8]. The results of an ongoing randomised controlled trial are awaited before use in clinical practice can be considered.

Moreover, in the context of pyridoxine-dependent epilepsy, innovative strategies like anti-sense therapies have been recently proposed, resulting in normal splicing processes and functional proteins in a sequence and dose-dependent fashion [9]. Future studies are warranted to ascertain the clinical efficacy of such a promising approach.

### Second-generation antiepileptic drugs

The use of some of the “newer” already available antiepileptic drugs has become wider in clinical practice, although off-label, and their results are described in case series. The main examples include levetiracetam and topiramate, which display favourable pharmacokinetic profiles and alternative mechanisms of action which could positively affect tolerability and efficacy in the neonatal period [10]. The authors included levetiracetam in the present algorithm, but, presumably due to the relative paucity of clinical data, suggest its use as the preferred second-line approach, and not as a possible first-line or directly after administration of the first phenobarbital bolus. We agree that levetiracetam use seems promising but, unfortunately, clinical data are still insufficient to recommend its use as a first-line drug.

For the time being, data on topiramate pharmacokynetics in normothermic neonates are scanty. This issue, together with the unavailability of an intravenous formulation [10] has resulted in a limitation of its use and inclusion in treatment algorithms, despite promising results in small clinical studies [11]. Moreover, as an important goal of timely control of seizures in neonates consists of improving long-term outcome, the existence of neuroprotective effects, demonstrated in experimental animal models for both these drugs [12-15], could be regarded as a relevant adjunctive criterion to guide drug choice and prioritisation.

### Therapeutic approach in metabolic epilepsies

In their systematic review, Slaughter and colleagues decided not to directly address the issue of seizures due to metabolic disorders, thus excluding articles with a primary focus on these conditions. They nevertheless include consideration of a pyridoxine trial in their treatment algorithm after second-line medications failure [3]. We suggest careful diagnostic work-up for early onset genetic-metabolic epileptic encephalopathies, with special care not to miss diagnosis whenever a disease-specific treatment is available [16].

### Therapeutic approach to preterm newborns

Furthermore, the issue of a possible impact of gestational age was not addressed, reflecting both the lack of clinical trials on this topic and the current clinical practice, which does not distinguish between term and preterm neonates [17], even if differences in pharmacokinetics, hepatic and renal function [10] and maturation of central nervous system could affect efficacy and reduce tolerability [10,18] in the preterm infant. It is advisable for future studies to address this issue in order to design different treatment algorithms in these two cohorts of patients and define adjusted dosages according to gestational age, if proven necessary.

### Therapeutic approach to hypothermic newborns

Among term newborns with moderate-to-severe hypoxic-ischemic encephalopathy, therapeutic hypothermia is now posing additional questions regarding neonatal seizures therapeutic options. Detailed data on pharmacodynamics and pharmacokinetics of antiepileptic drugs in hypothermic newborns are needed to enable correct prescription, and have been recently investigated in papers focusing on phenobarbital [19], lidocaine [20] and topiramate [21] administration. In addition, the existence of additive or synergistic neuroprotective effects has been demonstrated in both animals [22] and human newborns [23] for phenobarbital, whereas data regarding topiramate have been gathered in rodents in the context of ischemic stroke [24]. Nevertheless, in our opinion, the existence of common pathophysiological mechanisms leading to brain damage in the two conditions justifies the scientific interest around topiramate administration during hypothermia. Therefore therapeutic algorithms might conveniently be adapted or differentiated according to the condition of normothermia versus hypothermia.

### Therapeutic approach in limited-resource settings

Finally, a great effort has been made to improve medical management of neonatal seizures in different clinical settings, according to varying professional, instrumental and therapeutic resources [25,26]. Protocols proposed for developing Countries rely on traditional drugs [26], and their accessibility has been strongly promoted [25]. In this respect, phenobarbital remains an essential anticonvulsant due to easiness of administration, wider availability and cheapness compared to phenytoin [25].

## Conclusions

In summary, although phenobarbital still remains the first-line choice, the use of drugs with alternative mechanisms of action and the avoidance of neurotoxic compounds should be preferred, with the potential to positively impact on neurodevelopmental outcome. Hopefully innovative, efficacious treatment approaches will become available in the near future, as recent studies have mostly focused or will more likely focus on newer antiepileptic drugs.

Moreover, the establishment of dedicated protocols for preterm versus term and for normothermic versus hypothermic babies and continuous implementation of protocols for poor-resource settings is warranted to improve timely control of neonatal seizures.
